# Longitudinal molecular analysis of clinical and fecal *Escherichia coli* isolates at a Veterans Affairs Medical Center in Minnesota, USA, 2012–2019

**DOI:** 10.3389/fmicb.2024.1409272

**Published:** 2024-06-03

**Authors:** Connie Clabots, Paul Thuras, James R. Johnson

**Affiliations:** ^1^Minneapolis VA Health Care System, Minneapolis, MN, United States; ^2^Department of Psychiatry, University of Minnesota, Minneapolis, MN, United States; ^3^Department of Medicine, University of Minnesota, Minneapolis, MN, United States

**Keywords:** *Escherichia coli*, molecular epidemiology, ST131, ST1193, fluoroquinolone resistance, recurrent infections, intestinal colonization, clinical microbiology

## Abstract

**Introduction:**

Extraintestinal *Escherichia coli* infections represent a growing public health threat, However, current studies often overlook important factors such as temporal patterns of infection, phylogenetic and clonal background, or the host gut *E. coli* population, despite their likely significance.

**Methods:**

In this study, we analyzed >7000 clinical *E. coli* isolates from patients at the Minneapolis Veterans Affairs Health Care System (2012–2019), and concurrent fecal *E. coli* from uninfected veterans. We assessed phylogenetic group distribution, membership in selected sequence types (STs), and subsets thereof—including the pandemic, resistance-associated ST131-*H*30R, and ST1193 lineages—and strain type, as defined by pulsed-field gel electrophoresis. We then analyzed these features alongside the temporal patterns of infection in individual hosts.

**Results:**

The *H*30R lineage emerged as the leading lineage, both overall and among fluoroquinolone-resistant isolates, with ST1193 following among fluoroquinolone-resistant isolates. Recurrences were common, occurring in 31% of subjects and 41% of episodes, and often multiple and delayed/prolonged (up to 23 episodes per subject; up to 2655d post-index). Remarkably, these recurrences typically involved the subject’s index strain (63% of recurrences), even when affecting extra-urinary sites. ST131, *H*30R, ST1193, and fluoroquinolone-resistant strains generally caused significantly more recurrences than did other strains, despite similar recurrence intervals. ST131 strain types shifted significantly over the study period. Infection-causing strains were commonly detectable in host feces at times other than during an infection episode; the likelihood of detection varied with surveillance intensity and proximity to the infection. *H*30R and ST1193 were prominent causes of fecal-clinical clonal overlap.

**Discussion:**

These findings provide novel insights into the temporal and clonal characteristics of *E. coli* infections in veterans and support efforts to develop anti-colonization interventions.

## Introduction

*Escherichia coli*, a major extraintestinal pathogen, causes tremendous morbidity, mortality, and increased healthcare costs (Patton et al., 1991; Foxman, 2002; Russo and Johnson, 2003; Bonten et al., 2021). Its clinical and economic impacts are growing due to the rising prevalence of antimicrobial resistance, including advanced agents (O’Neill, 2014).

Within this phylogenetically diverse species, antimicrobial resistance tends to be clonally linked, as does the ability to cause extraintestinal infections (Russo and Johnson, 2000; Tchesnokova et al., 2017; Manges et al., 2019; Tchesnokova et al., 2020). Most humans are colonized intestinally with one or multiple *E. coli* strains and can carry them for varying periods, usually without experiencing disease (Bettelheim et al., 1972; Le Gall et al., 2007). Specific *E. coli* lineages, or clones, tend to predominate among clinical isolates, due to both their prominence in the gut reservoir and their intrinsic disease-causing ability (Caugant et al., 1983). Clonal sweeps through the population can shift the clonal structure of the host’s gut *E. coli*, thereby influencing both the likelihood of infection and whether an infection is antimicrobial-resistant (Manges et al., 2006; Smith et al., 2008; Tchesnokova et al., 2020).

Starting around 2000, a new pathogenic *E. coli* lineage, known as the *H*30R subclone within sequence type (ST) 131 (hereafter referred to as *H*30R), emerged as a leading cause of antimicrobial-resistant *E. coli* infections (Johnson et al., 2013; Banerjee and Johnson, 2014). Its multi-resistance phenotype included resistance to fluoroquinolones and extended-spectrum cephalosporins (ESCs), the latter mainly within the ST131-*H*30Rx subset of *H*30R (Price et al., 2013). Proposed contributors to the *H*30R emergence include resistance to heavily used antimicrobial agents, greater virulence, and greater persistence in the host’s gut (Ben Zakour et al., 2016; McNally et al., 2016; Overdevest et al., 2016; Mohamed et al., 2020; Tchesnokova et al., 2020; Johnson et al., 2022). More recently, another emergent multi-resistant lineage, ST1193, has joined ST131-*H*30R as a pandemic pathogen of concern, in some instances rivaling ST131-*H*30R for prevalence (Wu et al., 2017; Tchesnokova et al., 2018; Johnson et al., 2019; Kidsley et al., 2020; Pitout et al., 2022; Wyrsch et al., 2022).

The emergence of problematic strains like *H*30R and ST1193 has aggravated the vexing problem of recurrent *E. coli* infections, which has been studied mainly in the context of urinary tract infection (UTI), the most common type of *E. coli* infection (Stamm et al., 1991). Recurrent UTIs can be caused by the host’s previous UTI strain or a different strain (Kunin, 1962; Brauner et al., 1992; Ikäheimo et al., 1996; Karkkainen et al., 2000; Jantunen et al., 2002; Thänert et al., 2019). Same-strain recurrences are presumably due to either endogenous relapse from a persisting internal focus or the reintroduction of the strain from an external reservoir, such as the host’s gut or vagina, or from a colonized sex partner or other household members (Al-Wali et al., 1989; Foxman et al., 1996; Ulleryd et al., 2015; Johnson et al., 2016). Biofilm-embedded bacterial colonies within uroepithelial cells are a recently recognized potential explanation for some same-strain recurrent UTIs, analogous to classic factors such as infected calculi and chronic prostatitis (Mulvey et al., 2001; Rosen et al., 2007). These urinary tract-specific mechanisms, however, could not explain the same-strain recurrences outside of the urinary tract.

In this study, we capitalized on the availability of *E. coli* clinical isolates from veterans, as provided by the Minnesota Veterans Affairs Health Care System (MVAHCS) clinical microbiology laboratory, and uninfected veterans willing to serve as fecal donors, to observationally address multiple questions about *E. coli* infections in veterans. Specifically, we sought to define the clonal composition of the clinical *E. coli* population, to assess for clonal shifts over time, and to define the temporal distribution of infections in relation to the clonal background, site of infection, and gut *E coli* population, giving special attention to *H*30R and ST1193.

## Materials and methods

### Clinical isolates

From April 2012 to November 2019, the MVAHCS clinical microbiology laboratory provided to the research laboratory all its clinical *E. coli* isolates, as recovered from diverse clinical specimens from veterans, with associated antimicrobial susceptibility results. Species identification and susceptibility testing were done by the clinical laboratory using a bioMerieux VITEK® instrument. Susceptibility interpretations used current Clinical and Laboratory Standards Institute breakpoints. In the research laboratory, isolates were freshly passaged on BD BBL™ Columbia Agar with 5% sheep blood (Becton Dickinson, Franklin Lakes, NJ), then stored at −80^0^C in 20% glycerol until processed further. Isolates resistant to ciprofloxacin were considered fluoroquinolone-resistant; those resistant to ceftriaxone and/or ceftazidime were considered ESC-resistant.

### Fecal isolates

As described elsewhere (Johnson et al., 2019, 2022), from 2014 to November 2018, fecal *E. coli* isolates were recovered from self-collected fecal swabs provided by veterans at MVAHCS according to an Institutional Review Board-approved protocol. Veterans were recruited by sending invitations for study participation to all newly discharged MVAHCS inpatients and randomly selected outpatients. Consenting veterans collected fecal swabs and mailed them at room temperature in a commercial transport medium to the research laboratory. Then, the swabs were plated to Gram-negative selective media with and without ciprofloxacin (4 mg/L) for overnight incubation at 37^o^C. Indole-positive, citrate-negative colonies with a characteristic *E. coli* morphology were regarded presumptively as *E. coli*.

### PCR-based testing

All isolates were tested using established multiplex PCR-based assays to identify seven major *E. coli* phylogroups (A, B1, B2, C, D, E, and F) (Clermont et al., 2012); nine clonal subsets within group B2 (ST complex [STc] 12, STc14, STc73, STc95, STc127, STc131, STc141, STc144, and STc372) (Clermont et al., 2014), three within group D (STc31, ST69, and ST405), and one within group F (ST48); and two subclones within ST131 (ST131-*H*30 and its subset ST131-*H*30Rx) (Johnson et al., 2017, Table 1). Ciprofloxacin-resistant *H*30 isolates were regarded as representing the ST131-*H*30R subclone. All PCR testing was done in duplicate, with appropriate internal positive and negative controls.

**Table 1:** Characteristics of clinical *Escherichia coli* isolates from veterans, 2012–2019.

	Prevalence of characteristic, no. of isolates (column %)				
Characteristic^a^	Total (*n* = 7076)	FQ-S^b^ (*n* = 5199)	FQ-R^b^ (*n* = 1877)	ESC-R^b^ (*n* = 466)	*p*-value^c^, FQ-S^a^ vs. FQ-R^a^
Group A	407 (6)	284 (5)	123 (7)	45 (11)	0.09
Group B1	473 (7)	423 (8)	50 (3)	18 (4)	<0.001
Group B2	5215 (74)	3763 (72)	1452 (77)	303 (65)	<0.001
ST131	1538 (22)	335 (6)	1203 (64)	249 (53)	<0.001
ST131-*H*30	1194 (17)	36 (0.7)	1158 (62)	225 (48)	<0.001
ST131-*H*30Rx	271 (4)	1 (0.02)	270 (14)	120 (26)	<0.001
STc12	410 (6)	410 (8)	0 (0)	5 (1)	<0.001
STc14	321 (5)	119 (2)	202 (11)	18 (4)	<0.001
ST1193	202 (3)	0 (0)	202 (11)	18 (4)	<0.001
STc73	735 (10)	731 (14)	4 (0.3)	6 (1)	<0.001
STc95	608 (9)	584 (11)	24 (1.3)	3 (0.6)	<0.001
STc127	666 (9)	662 (13)	4 (0.2)	4 (0.9)	<0.001
STc141	170 (2)	170 (3)	0 (0)	1 (0.2)	<0.001
STc144	74 (1)	74 (1)	0 (0)	1 (0.2)	<0.001
STc372	86 (1)	85 (2)	1 (0.1)	7 (1)	<0.001
STc not detected^d^	489 (7)	479 (9)	10 (0.5)	6 (1)	<0.001
STc indeterminate^e^	117 (2)	113 (2)	4 (0.2)	3 (0.6)	<0.001
Group C	56 (0.8)	38 (0.7)	18 (1)	12 (3)	0.36
Group D	696 (10)	543 (10)	153 (8)	68 (10)	0.004
ST31 (O15)	29 (0.4)	26 (0.5)	3 (0.2)	5 (1)	0.06
ST69 (CGA^a^)	269 (4)	222 (4)	47 (3)	4 (1)	<0.001
ST405	97 (1.4)	48 (1)	49 (3)	29 (6)	<0.001
Group E	43 (0.6)	43 (1)	0 (0)	1 (0.2)	<0.001
Group F	153 (2)	82 (2)	71 (4)	16 (3)	<0.001
ST648	44 (0.6)	5 (0.1)	39 (2)	12 (3)	0.002
SAM resistance	2718 (38)	1458 (28)	1260 (67)	378 (81)	<0.001
TZP resistance	217 (3)	103 (2)	114 (6)	43 (9)	<0.001
KZ resistance	733 (10)	244 (5)	489 (26)	460 (99)	<0.001
CAZ resistance	395 (6)	87 (2)	308 (16)	375 (80)	<0.001
CRO resistance	466 (7)	99 (2)	367 (20)	466 (100)	<0.001
IMP resistance	4 (0.06)	1(0.02)	3 (0.2)	2 (0.4)	0.06
ERT resistance	8 (0.1)	4 (0.1)	4 (0.2)	4 (1)	0.22
CN resistance	578 (8)	178 (3)	400 (21)	174 (37)	<0.001
CIP resistance	1878 (27)	0 (0)	1878 (100)	367 (79)	n.a.^f^
SXT resistance	1567 (22)	645 (12)	922 (49)	268 (58)	<0.001
F resistance	350 (5)	168 (3)	182 (10)	70 (15)	<0.001

^a^Group, phylogenetic group; ST, sequence type; STc, sequence type complex; CGA, clonal group A; SAM, ampicillin-sulbactam; TZP, piperacillin-tazobactam; KZ, cefazolin; CAZ, ceftazidime; CRO, ceftriaxone; IMP, imipenem; ERT, ertapenem; CN, gentamicin; CIP, ciprofloxacin; SXT, trimethoprim-sulfamethoxazole; F, nitrofurantoin. ST131-*H*30 is a subset of ST131; ST131-*H*30Rx is a subset of ST131-*H*30. ^b^FQ-S, fluoroquinolone-susceptible; FQ-R, fluoroquinolone-resistant; ESC-R, extended-spectrum cephalosporin-resistant. ^c^*p*-values by a two-tailed Fisher’s exact test. ^d^STc not detected, group B2 STc PCR yielded no result. ^e^STc indeterminate, group B2 STc PCR yielded uninterpretable results. ^f^n.a., not applicable (0% vs. 100% by definition).

### PFGE analysis

Selected isolates—i.e., all ST131 isolates, and systematically or randomly selected representatives of other subsets relevant to the particular subproject (as described in detail below)—underwent genomic profiling using *Xba*I pulsed-field gel electrophoresis (PFGE) according to the PulseNet protocol (Ribot et al., 2006). Pulsotypes were defined at 94% profile similarity based on computer-assisted Dice coefficient analysis of banding patterns within BioNumerics (Applied Maths) (Johnson et al., 2012). Newly determined profiles were compared digitally with an existing large private profile library containing, at last count, 9300 profiles and 2575 pulsotypes (Johnson et al., 2012). Isolates from a given subject were regarded as isoclonal in comparison with one another if they exhibited the same pulsotype and as heteroclonal if they differed for pulsotype, phylogenetic group, and/or ST.

### Definitions

An episode was a set of one or more clinical isolates from a given subject within a defined time interval; the interval was selected empirically based on observed temporal patterns of isolate occurrence, as described below. A subject’s first isolate in the clinical collection was considered their index isolate, and subsequent isolates were considered repeat isolates. A subject’s first episode was considered their initial episode, and subsequent episodes were considered repeat episodes. Within an episode, the first isolate was considered the episode’s initial isolate and was used in subsequent analyses to represent the episode. (A subject’s index isolate was, thus, the initial isolate of the subject’s initial episode.) Episodes could comprise single or multiple isolates (single-isolate and multi-isolate episodes), as recovered from single or multiple specimens, whereas a given specimen could yield one or multiple isolates (single-isolate and multi-isolate specimens).

### Assessment for within-episode isoclonality and clonal persistence

To assess for clonality among isolates within a given multi-isolate episode we used a hierarchical approach to analyze the 1115 total isolates from all 534 multi-isolate episodes (534 initial isolates and 581 subsequent isolates). PFGE was used to compare same-episode isolates if they were indistinguishable according to phylogenetic group and fluoroquinolone phenotype.

To assess for clonal persistence over time in a given subject and predictors thereof, we performed clonal analysis on each episode’s initial isolate for all episodes from 115 subjects with multiple episodes. The study included 10 randomly selected subjects per number of repeat episodes, ranging from two to eight total episodes (60 subjects and 350 isolates), as well as all 45 subjects with 9–24 total episodes (554 isolates), resulting in an overall total of 904 isolates.

We next assessed phylogenetic background and fluoroquinolone resistance as predictors of the number of (isoclonal) episodes a particular strain caused in a given subject. For this, we identified all sets of isoclonal repeat episodes, and all clonally unique (i.e., singleton) episodes, among the above pulsotyped isolates (*n* = 521 sets or singletons).

From the 521 total isoclonal sets or singletons, we chose 321 for analysis. Specifically, we used all 135 identified clonal singletons from subjects with multiple episodes (135 isolates), 32 randomly selected clonal repeat sets per number of repeat episodes for sets with from two to four isoclonal episodes (total: 96 sets, 288 representative isolates), and all 90 sets with five to 19 isoclonal episodes each (total: 90 sets and 674 representative isolates). We supplemented this collection with 200 (inherently clonal singleton) isolates from subjects with a single episode.

### Statistical methods

For some analyses, isolate pairs were binned by inter-isolate time interval into groups of approximately 40 each. For other analyses, the number of isoclonal isolates (i.e., episodes) per set was used as a continuous outcome variable. Comparisons of proportions were tested using Fisher’s exact test (two-tailed). Comparisons involving continuous variables were tested using the Mann–Whitney test (two-tailed). Negative binomial regression, as implemented using R, was used to investigate the effects on number of isolates (episodes) per isoclonal set for those predictor variables that were significant in univariate analyses. Negative binomial regression was used because the isolate (episode) count variable was an over-dispersed Poisson distribution. Regression results were expressed as incidence rate ratios (IRRs) and 95% confidence intervals (CIs). Throughout, the criterion for statistical significance was *p* < 0.05, with *p*-values from 0.05 to 0.10 considered to reflect borderline statistical significance. Given the exploratory nature of the analyses, no adjustment was made for multiple comparisons.

## Results

### Study population

From April 2012 to November 2019, the MVAHCS clinical microbiology laboratory recovered 7076 total *E. coli* isolates from 3851 total veterans. The most common source specimen type was urine (5535, 78%), followed by wound (676, 10%), and blood (404, 6%), with diverse other specimen types (e.g., fluid, tissue, abscess, and sputum) accounting for <2% of isolates each. Individual subjects contributed from one isolate (41% of subjects) to 24 isolates each (median number per subject, 2). Most specimens yielded only one isolate, but some (*n* = 159) yielded two or three isolates according to the clinical laboratory’s criteria.

### Isolate characteristics

The 7076 study isolates were phylogenetically diverse (Table 1). Group B2 predominated (74%), with its leading subset, ST131 (22%), represented mainly by *H*30 (17%). Ninety-seven percent of *H*30 isolates were fluoroquinolone-resistant, i.e., represented *H*30R (16%). *H*30R accounted for 62% of all fluoroquinolone-resistant isolates and included the extensively resistant *H*30Rx subclone (4%). *H*30R was more abundant than any non-ST131 STc within group B2, including the three leaders (STc73, STc93, and STc127: 9–10% each), and than any non-B2 phylogenetic group (A, B1, C, D, E, and F) (≤10% each).

Of the 7076 isolates, 22% were fluoroquinolone-resistant (Table 1). All but three of the studied phylogenetic subsets were significantly associated (positively or negatively) with fluoroquinolone resistance. After *H*30R, the next most frequent fluoroquinolone-resistant lineage was ST1193, within ST14c (11% of fluoroquinolone-resistant isolates). The clinical isolates’ diverse other resistance phenotypes, ranging in frequency from 0.06% (imipenem) to 38% (ampicillin-sulbactam) (Table 1), were all significantly or borderline significantly more prevalent among fluoroquinolone-resistant isolates.

Additionally, 7% of isolates were ESC-resistant (Table 1). Approximately half of the ESC-resistant isolates were from group B2, mainly from ST131-*H*30, split between *H*30-Rx and *H*30 non-Rx. The rest were distributed broadly; after ST131-*H*30, the leading lineages were group A (11%), group D (10%), ST405 (6%), and group B1 and ST1193 (4% each). The ESC-R isolates had an even larger co-resistant fraction than the FQ-resistant isolates for all agents except ciprofloxacin.

### Temporal distribution of clinical isolates by subject

**Figure 1:**
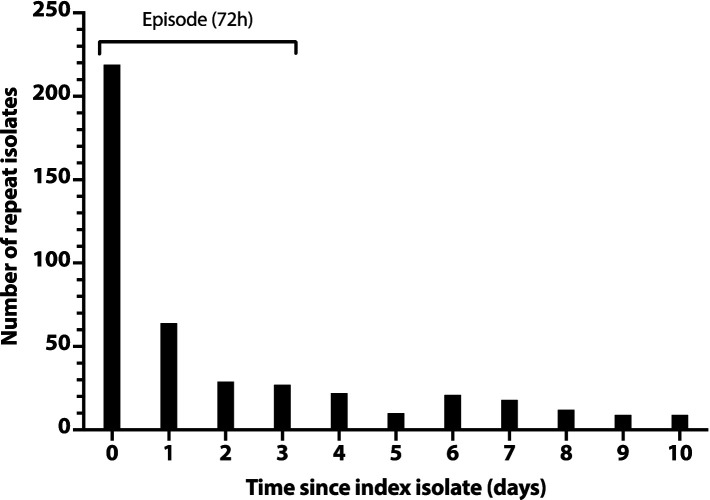
Number of repeat *Escherichia coli* clinical isolates per day after a subject’s index isolate. Number of repeat isolates per day (*y*-axis) is the daily total number of post-index isolates across all subjects, counting from each subject’s day 0 index isolate. Repeat isolates shown on day 0 are additional isolates from the same day as the subject’s index isolate.

To rationally de-duplicate the study isolates, we assessed for temporal clustering that might indicate the typical duration of an infection episode. We first created a histogram with the total number of additional isolates per day for the first 10 days after a subject’s index isolate (Figure 1). This showed an initial steep drop in daily isolate numbers (days 0–2), followed by an abrupt plateau (days 2–3), then a slower drop, with minor day-to-day variation (days 4 and thereafter). This tentatively supported the use of a 3-day (72 h) interval to define an episode.

**Figure 2:**
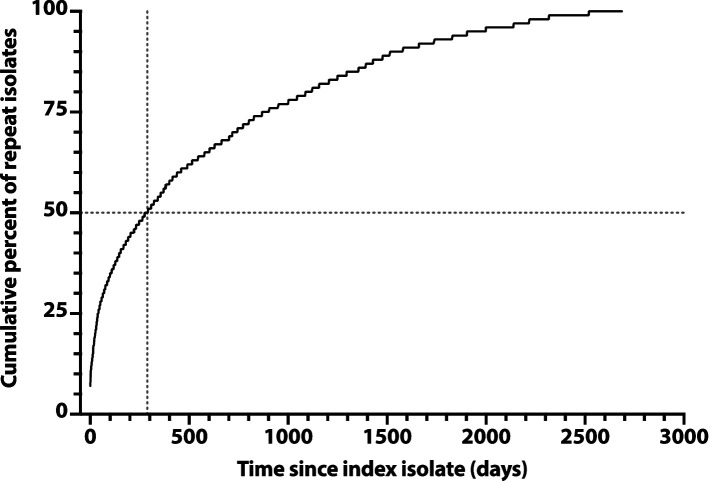
Saturation curve for the cumulative number of repeat *Escherichia coli* clinical isolates over time after an index isolate. Cumulative percent values (*y*-axis) are based on 3225 total repeat isolates, as recovered over 2,686 days post-index (*x*-axis) from 1176 veterans. Vertical dashed line: time to reach 50% accrual of repeat isolates (indicated by horizontal dashed line).

To assess later time points, we constructed a saturation curve showing the cumulative percent of repeat isolates accruing over time after a subject’s index isolate (Figure 2). The curve rose smoothly and steadily, with a progressively decreasing slope, reaching the 50% point at day 285, with no obvious inflection point to suggest an alternative natural episode duration.

Accordingly, we adopted 72 h as the operational duration of an episode. The rare isolates that were separated by >72 h from one another, but by ≤72 h from one or more intervening isolates from the same subject, were regarded as representing the same episode.

Using this definition, the study population represented 6495 total episodes, including 5961 (92%) single-isolate episodes and 534 (8%) multi-isolate episodes. Analysis of the 1115 isolates from the 534 multi-isolate episodes showed that the likelihood of isoclonality between an episode’s initial isolate and another isolate from the same episode was 90% overall, which rose to 93% if the isolates’ ciprofloxacin phenotypes matched, versus 0% if they conflicted. In the majority (54%) of the 534 multi-isolate episodes, the isolates were derived from multiple specimen types, with varying combinations. The most common combinations were urine plus blood (32%), “other” plus blood (5%), and urine plus wound (5%).

### Repeat episodes

Of the 3851 source subjects for the clinical isolates, 2675 (69%) contributed 1 episode each, whereas 1176 (31%) contributed from 2 to 24 (median, 2) episodes, for a total of 2644 repeat episodes (41% of 6495 total episodes). Repeat episodes occurred from 0.1 to 89.5 months (median, 4.4 months) after the subject’s initial episode.

**Figure 3:**
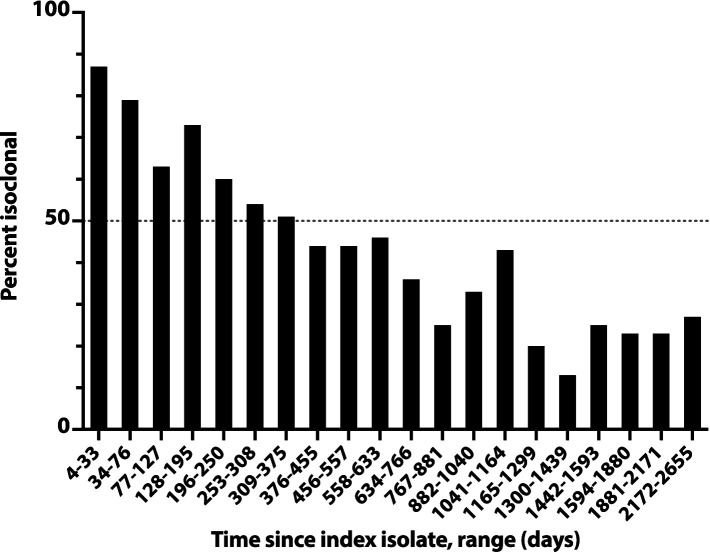
Time dependence of isoclonality between index *Escherichia coli* clinical isolate and same-host repeat isolates. Isoclonality with the subject’s index isolate was assessed by pulsed-field gel electrophoresis for 789 repeat isolates from 115 systematically selected veterans with repeat isolates. Based on time since the subject’s index isolate (*x*-axis), isolates were binned into 20 groups of 38–41 isolates each, and each group’s aggregate percent isoclonal with the index isolate (*y*-axis) was calculated. Horizontal dashed line, 50% isoclonal point.

The likelihood of isoclonality between a subject’s index isolate and a subsequent episode’s initial isolate declined slowly over time, reaching 50% only at 376d-455d (i.e., 12–15 months) post-index, and remained >20% even at 2172d-2655d (71–87 months) post-index (Figure 3). The median post-index interval was only 36% as long for isoclonal as for heteroclonal repeat isolates (339d vs. 944d: *p* < 0.001, Mann–Whitney test).

**Figure 4:**
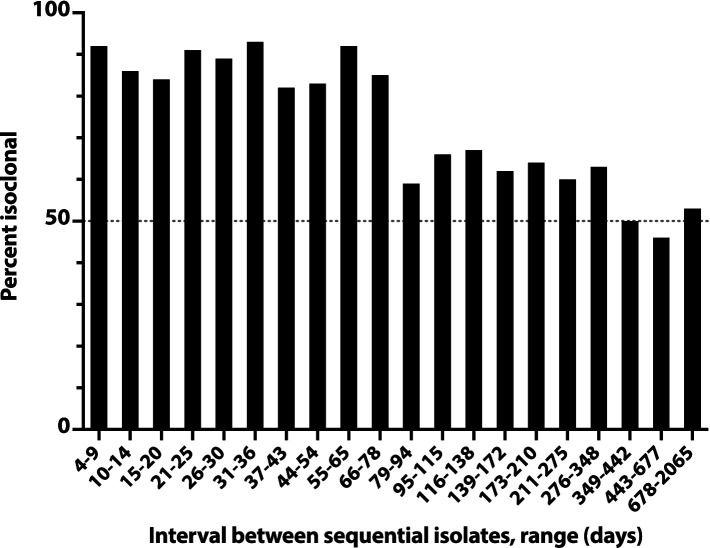
Time dependence of isoclonality between sequential same-host *Escherichia coli* clinical isolates. Isoclonality between sequential-episode, same-host *E. coli* clinical isolates was assessed by pulsed-field gel electrophoresis for 789 repeat isolates from 115 systematically selected veterans. Based on interval, isolate pairs were binned into 20 groups of 36–44 pairs each, and each group’s aggregate percent isoclonal (*y*-axis) was calculated. Horizontal dashed line, 50% isoclonal point.

A similar analysis of all pairs of sequential initial isolates from these subjects likewise showed an inverse relationship between inter-isolate interval and likelihood of within-pair isoclonality, which remained ≥50% even for the longest inter-isolate intervals observed (e.g., 678d-2065d, 22–70 months) (Figure 4). The inter-isolate interval was only 35% as long for isoclonal as for heteroclonal pairs (median, 56d vs. 159d: *p* < 0.001, Mann–Whitney test).

Overall, 43% of the selected repeat isolates were isoclonal with the subject’s index isolate (Figure 3). Extrapolations based on relative subset size and percent isoclonal per subset indicated that, among all repeat isolates, the total isoclonal-with-index fraction was actually 68%. Analogous calculations indicated that 73% of the selected repeat isolates were isoclonal with the immediately preceding isolate, and that among all repeat isolates, the total isoclonal-with-preceding-isolate fraction was actually 76%.

Of the 340 repeat episodes for which the initial isolate was isoclonal with the subject’s index isolate, most (294, 86%) involved a urine source for both the index and repeat isolate. A minority, however (46, 14%), paired a non-urine isolate with a urine isolate or involved only non-urine isolates. These isoclonal non-urine isolates were from diverse sources, including abscess, blood, bone, drainage, fluid, gallbladder, rectal, sputum, tissue, and wound. The percent isoclonal was similar whether the paired isolates were from the same or different specimen types (i.e., 89% vs. 86%: *p* > 0.05).

### Strain characteristics as correlates of recurrence interval

Using this same set of repeat isolates, we compared the interval between sequential isolates (isolate pairs) with isolate characteristics, including phylogenetic background and within-pair isoclonality (789 total pairs: 73% isoclonal, 27% heteroclonal). The median recurrence interval, which overall was 78 days (range, 4d-2065d), was only 35% as long for the 576 isoclonal pairs (median, 56d [range, 4d-2065d]) as for the 213 heteroclonal pairs (median, 160d [range, 7d-1846d]) (*p* < 0.001, Mann–Whitney test). By contrast, with two exceptions (described below), the recurrence interval did not vary significantly in relation to phylogenetic group, ST, fluoroquinolone phenotype, or whether the paired isolates shared or differed for these characteristics (not shown). The exceptions, which were barely statistically significant, were (i) a shorter recurrence interval for isoclonal group A isolates (10 pairs: median interval, 45 days) than for isoclonal group C isolates (four pairs: median interval, 90.5 days) (*p* = 0.02), and for fluoroquinolone-resistant isoclonal isolates (59 pairs: median interval, 56 days) than for fluoroquinolone-susceptible isoclonal isolates (60 pairs: median interval, 65 days) (*p* = 0.04).

### Strain characteristics as correlates of the number of episodes

We next assessed phylogenetic background and fluoroquinolone resistance as predictors of the number of episodes a given strain caused in a given subject (Table 1). This was done using a systematically selected sample of subjects with single or multiple episodes, who collectively yielded 521 sets of same-subject isoclonal isolates, each set representing from one to 19 episodes.

Of the studied phylogenetic variables, those most closely associated with the number of episodes were all from group B2. First was ST1193 (median, 6 episodes), followed by STc14 (median, 5 episodes) (Table 2). The next most closely associated were *H*30 (median, 4 episodes), ST131 (median, 3 episodes), and non-*H*30 ST131 strains (median, 3 episodes). By contrast, group B2 STs and ST complexes other than ST1193 and ST131 were unassociated with the number of episodes, whereas B2 strains of undefined STc exhibited a negative association. Accordingly, group B2 overall exhibited a weak positive association with the number of episodes (median, 1 episode; *p* = 0.04). Except for group B1, which exhibited a significant negative association with the number of episodes, none of the non-B2 phylogenetic groups or studied STs was associated with the number of episodes. Fluoroquinolone resistance was also highly significantly associated with number of episodes (median, 4 episodes) (Table 2).

**Table 2:** Number of isoclonal episodes in relation to phylogenetic background and fluoroquinolone phenotype.

	No. isoclonal episodes per set^a^						
	Mean		Median		75th percentile		
Phylogenetic or resistance category^b,c^ (no. sets, % of 521)	In category	Others	In category	Others	In category	Others	*p*-value^b^
Group B1 (38, 7%)	1.16	2.59	1	1	1	4	<0.001
Group B2 (370, 71%)	2.62	2.17	1	1	4	2	0.04
Group B2, ST131 (123, 24%)	3.94	2.04	3	1	5	2	<0.001
ST131-*H*30 (100, 19%)	3.94	2.14	4	1	5	2	<0.001
ST131, non-*H*30 (23, 4%)	2.66	2.42	3	1	5	3	0.006
Group B2, non-ST131-*H*30 (270, 52%)	2.13	2.80	1	1	2.25	4	0.007
Group B2, non-ST131 (247, 47%)	1.96	2.96	1	1	2	4	<0.001
Group B2, STc14 (16, 3%)	5.13	2.40	5	1	7.75	3	<0.001
Group B2, ST1193 (14, 3%)	5.71	2.39	6	1	8.25	3	<0.001
Group B2, STc undefined (29, 6%)	1.45	2.55	1	1	1	3	0.02
Fluoroquinolone resistance (150, 29%)	4.25	1.78	4	1	6	1	<0.001

^a^Sets comprised all initial *E. coli* isolates (one per episode) from a given subject that exhibited the same pulsotype. Subjects could contribute one or multiple sets each. ^b^Categories shown are those that yielded *P* < 0.05 (using the Mann–Whitney *U* test) for the comparison of sets in that category vs. all other sets combined for the number of isolates (episodes) per set. ^c^Phylogenetic categories that yielded *P* ≥ 0.05 for the comparison of sets in that category vs. all other sets included (no. sets in category, % of 521): STc12 (13, 2%), STc73 (37, 7%), STc127 (35, 7%), STc141 (11, 2%), STc144 (6, 1%), STc372 (7, 1%), group A (34, 7%), group C (5, 1%), group D (58, 11%), group E (2, 0.4%), group F (14, 3%).

Because multiple variables correlated with the number of episodes, we used negative binomial regression to identify, among the significant univariable predictor variables, those variables with independent predictive power. The strongest multivariable predictor of number of episodes (IRR [95% CI]) was fluoroquinolone resistance (IRR 2.38 [1.82, 3.11]), followed by ST131 (IRR 1.99 [1.44, 2.77]). ST1193 also had a high IRR, but the confidence interval included 1.0, rendering it non-significant (IRR 2.40 [0.55, 16.77]). By contrast, both ST131-*H*30 (IRR 0.48 [0.32, 0.72]) and group B1 (IRR 0.56 [0.38, 0.82]) were significant negative predictors.

### Temporal distribution of ST131 pulsotypes over the study period

The study’s pulsotyped ST131 clinical isolates (total, 1178; one per episode) represented 192 distinct PFGE types, which were segregated almost completely by fluoroquinolone phenotype; only nine types included both susceptible and resistant isolates. The pulsotypes varied widely in abundance, accounting for from >20% to <0.1% of the ST131 population each (Supplementary Table S1 and Table 3).

**Table 3:** Temporal dynamics of the most abundant unique-by-subject PFGE^a^ types among clinical ST131^b^ isolates, 2012–2019.

	Time period		
Isolate group	2012–2015	2016–2019	*p*-value^c^
Total unique^d^ ST131 isolates, no.	396	356	n.a.
FQ-S^e^ unique^d^ ST131 isolates, no. (% of total unique^d^ ST131 isolates)	91 (23)	112 (31)	0.01
FQ-R^e^ unique^d^ ST131 isolates, no. (% of total unique^d^ ST131 isolates)	305 (78)	244 (69)	0.01
Main 3 FQ-S PFGE types^f^, no. isolates (% of unique^d^ FQ-S ST131 isolates)	25 (27)	42 (38)	0.14
Main 3 FQ-S PFGE types^f^, no. isolates (% of total unique^d^ ST131 isolates)	25 (6)	42 (12)	0.01
Main 3 FQ-R PFGE types^f^, no. isolates (% of unique^d^ FQ-R ST131 isolates)	148 (49)	101 (41)	0.10
Main 3 FQ-R PFGE types^f^, no. isolates (% of total unique^d^ ST131 isolates)	148 (37)	101 (28)	0.01

^a^PFGE, pulsed-field gel electrophoresis. ^b^ST131, Escherichia coli sequence type 131. ^c^P values using Fisher’s exact test (two-tailed) for comparison of 2012–2015 vs. 2016–2019. ^d^Unique, includes only one representative of a given PFGE type per subject. ^e^FQ-S, fluoroquinolone-susceptible. FQ-R, fluoroquinolone-resistant. ^f^FQ-S and FQ-R PFGE types, PFGE types comprising mainly or exclusively FQ-S or FQ-R isolates. Main 3 FQ-S PFGE types: 1140, 1459, and 1464. Main 3 FQ-R PFGE types: 800, 968, and 1689.

Of the 192 PFGE types, 73 included one or more fluoroquinolone-susceptible isolates. The three most common of these types (1140, 1459, and 1464) accounted jointly for a significantly greater share of unique (by subject and PFGE type) ST131 isolates during the study’s second half than its first half (Table 3).

By contrast, 128 types included one or more fluoroquinolone-resistant isolates. The three most common of these types (800, 968, and 1689) accounted jointly for a significantly greater share of unique ST131 isolates during the study’s first half than its second half (Table 3). These contrasting temporal trends reflected both (i) a statistically significant overall temporal increase in the fluoroquinolone-susceptible fraction among unique ST131 isolates across the study period and (ii) non-significant relative increases or decreases, respectively, for the three main PFGE types among fluoroquinolone-susceptible and fluoroquinolone-resistant ST131 isolates (Table 4).

**Table 4:** Clonal overlap^a^ between fecal and clinical *Escherichia coli* isolates from 33 veterans.

					Pulsed-field gel electrophoresis (PFGE) types^b^	
Clinical- fecal overlap^a^	Subject	Fecal sam^c^, no.	Fecal iso^d^, no.	Clinical iso^d^, no.	Fecal (no. isolates per type)	Clinical (no. isolates per type)
Yes	1	5	6	1	*968* (1)*,**1260***^e^ (4), 1940 (1)	***1260*** ^e^ ***(1)***
	2	9	17	1	***1297***^f^ (9), 2025 (3), 2264 (3), 2265 (1), 2266 (1)	***1297*** ^f^ ***(1)***
	3	6	6	3	***1297***^f^ (6)	***1297*** ^f^ ***(3)***
	4	17	18	2	***808*** (17), 2289 (1)	***808*** (1)
	5	4	5	1	135 (1),***1943***^g^ (4)	***1943***^g^ (1)
	6	8	8	5	***968*** (1), *1067* (7)	***968*** (2)*, 1649 (3)*
	7	8	8	3	***968*** (8)	***968*** (3)
	8	11	15	2	1924 (1), 1959 (1),***1960***^i^ (11), 2106 (1), 2108 (1)	***1960***^i^ (2)
	9	1	3	3	**888**^h^ (1), 2417 (1), 2418 (1)	**888**^h^ (1), 2029 (2)
No	10	1	1	1	2419 (1)	997 (1)
	11	1	1	5	*1619* (1)	691 (1), *968* (2), 1558 (1)
	12	10	12	1	*1677*^i^ (10), 2268 (1), 2269 (1)	1902 (1)
	13	1	1	1	1218 (1)	857 (1)
	14	1	1	2	2425 (1)	2407 (1), 2414 (1)
	15	1	1	1	2342 (1)	2165 (1)
	16	1	1	1	2409 (1)	2413 (1)
	17	1	1	1	2341 (1)	2427 (1)
	18	1	1	1	1990 (1)	1938 (1)
	18	1	2	2	2419 (1), 2424 (1)	1140 (2)
	20	1	2	4	2422 (1), 2423 (1)	1773 (1), 2180 (1), 2245 (1), 2410 (1)
	21	1	2	1	607 (1), 2416 (1)	2187 (1)
	22	1	1	4	*2420* (1)	422 (1), 1588 (1), *2404* (1), 2405 (1)
	23	1	10	1	2138 (10)	2403 (1)
	24	1	1	4	1950 (1)	2412 (1), 2428 (3)
	25	11	18	5	1394 (6), *1689* (11), 2244 (1)	*800* (1), 2406 (4)
	26	1	1	3	2428 (1)	526 (3)
	27	1	1	1	1972 (1)	915 (1)
	28	13	12	3	*968* (6), *1689* (4), *1722* (2)	*560* (2), 1481 (1)
	29	1	1	6	1412 (1)	1373 (6)
	30	1	1	1	862 (1)	1595 (1)
	31	1	1	1	1464 (1)	128 (1)
	32	1	1	1	1464 (1)	351 (1)
	33	1	2	3	6 (1), 2415 (1)	1525 (2), 2102 (1)

^a^Clinical-fecal overlap with respect to PFGE types (irrespective of temporal correspondence). ^b^PFGE types are listed once per subject per specimen type (fecal, clinical). Boldface, clinical-fecal overlap type. Italics, fluoroquinolone-resistant. Underline, ST131. Italics plus underline, ST131-H30. ^c^Sam, samples. ^d^Iso, isolates. ^e^Type 1260: O15 clonal group (phylogroup D). ^f^Type 1297: ST1193 (phylogroup B2). ^g^Type 1943: ST405 (phylogroup D). ^h^Type 1960: ST648 (phylogroup F). ^i^Type 888: ST73 (phylogroup B2). ^j^Type 1677: ST69 (phylogroup D).

### Prevalence trends for ESC resistance, ST131 among ESC-resistant isolates, and H30Rx

Among the 6495 initial isolates, the ESC-resistant fraction varied by year from 5 to 8% (median, 6%). Despite no obvious temporal trend (by year, from 2012–2019: 6, 7, 5, 5, 6, 7, 8, 6%), the aggregate ESC-resistant fraction was higher during the second half of the study (2016–2019: 243/4373, 7%) than the first half (2012–2015: 168/3022, 5.6%) (*p* = 0.02). By contrast, among ESC-resistant initial isolates, the ST131 fraction varied by year from 44 to 64% (median, 56.5%), without no obvious temporal trend (by year, from 2012–2019: 57, 64, 59, 44, 56, 64, 47, 49%), and was statistically similar overall between the first and second half of the study (95/168 [57%], vs. 128/24 [53%]: *p* = 0.48). Similarly, among all initial isolates the *H*30Rx fraction varied by year from 3.1 to 5.3% (median 3.7%), with no obvious temporal trend (by year, from 2012 to 2019: 3.1, 4.5, 3.0, 3.8, 3.1, 3.6, 5.3, 4.1%), and was statistically similar overall between the first and second half of the study (109/303 [3.6%], vs. 140/3473, [4.0%]: *p* = 0.40).

### Correspondence between clinical and fecal *Escherichia coli* isolates in a given subject

In a concurrent longitudinal fecal surveillance study, 1125 veterans who were uninfected at the time of enrollment provided from one to 17 serial fecal samples each, collectively yielding 2356 distinct *E. coli* isolates. During clinical surveillance, 42 of the 1125 fecal surveillance subjects had one or more *E. coli* clinical isolates that were captured serendipitously by the primary study. Fecal samples from 33 (79%) of these 42 veterans yielded *E. coli*, for clonal comparison with the subject’s clinical *E. coli* isolate(s).

These 33 subjects each contributed from one to five distinct fecal *E. coli* strains, and from one to four distinct clinical *E. coli* strains (Table 4). Nine (24%) subjects had a clonal match between their fecal and clinical *E. coli* isolates. As compared to the 24 fecal surveillance subjects with fecal and clinical *E. coli* but no fecal-clinical overlap, the nine subjects with fecal-clinical overlap had significantly more fecal samples per subject (medians, 8 vs. 1: *p* < 0.001), more fecal *E. coli* isolates per subject (medians, 8 vs. 1: *p* < 0.001), and more distinct fecal *E. coli* strains per subject (medians, 2 vs. 1: *p* = 0.03) (Mann–Whitney *U* test). Additionally, their fecal surveillance period and dates of clinical isolate recovery more often overlapped or were separated by <60d (5/9 vs. 3/24: *p* = 0.02). By contrast with these fecal sampling differences, the two groups had comparable per-subject numbers of clinical isolates and distinct clinical strains (for both comparisons, *p* > 0.20).

The nine fecal-clinical overlap *E. coli* strains were disproportionately fluoroquinolone-resistant (8/9, 89%), whether in comparison with other fecal strains from the same subjects (2/15, 13%) (*p* < 0.001) or from the 24 fecal surveillance subjects with no fecal-clinical clonal overlap (6/24, 25%) (*p* = 0.002) (Table 4). *H*30 was prominent among the nine fecal-clinical overlap subjects, accounting for three of nine overlap strains, two of 15 unmatched fecal strains, and one of three unmatched clinical strains (Table 4). It was similarly prominent among the 24 subjects without fecal-clinical clonal overlap, accounting for five of six fluoroquinolone-resistant fecal *E. coli* strains and six of the eight fluoroquinolone-resistant clinical *E. coli* strains. ST1193, by contrast, accounted for two fluoroquinolone-resistant fecal-clinical overlap strains, but for no unmatched fecal or clinical strains. Each of the remaining fluoroquinolone-resistant strains represented an ST that occurred only once in these 33 subjects.

## Discussion

In this study, we analyzed 7076 consecutive clinical *E. coli* isolates from veterans at the MVAHCS (2012–2019), comparing their phylogenetic and clonal background, resistance phenotypes, and specimen types with temporal patterns of recurrence and, in a subset, the host’s fecal *E. coli* population. Our findings support five main conclusions. First, the recently emerged ST131-*H*30 lineage was the most abundant lineage overall among *E. coli* clinical isolates, accounting for most fluoroquinolone-resistant isolates (62%) and a plurality of all isolates (16%). Second, among fluoroquinolone-resistant isolates, *H*30 was followed in abundance by ST1193, an even-more-recently emerged lineage. Third, both *H*30 and ST1193 had a prominent fecal reservoir that likely led to infections. Fourth, recurrent infections were common, often multiple, usually same-strain (even years later), and involved diverse anatomical sites. Fifth, number of recurrences was greatest for ST131 (both *H*30 and non-*H*30), ST1193, and fluoroquinolone-resistant strains generally, despite similar recurrence intervals.

The first main finding, i.e., the prominence of ST131 and its *H*30R subclone provides further evidence of the exceptional epidemiological success, of this recently emerged lineage (Banerjee and Johnson, 2014). The basis for the rapid, widespread expansion of *H*30R since about 2000 remains speculative but may include a combination of resistance to widely used antimicrobial agents (especially fluoroquinolones and extended-spectrum cephalosporins), superior gut colonizing ability, and—although questionably—greater virulence (Croxall et al., 2011; Banerjee and Johnson, 2014; Petty et al., 2014; McNally et al., 2016; Overdevest et al., 2016; Stoesser et al., 2016; Johnson et al., 2022). The broad similarities between the present strain set and clinical *E. coli* collections reported from other locales and hosts (Croxall et al., 2011; Salipante et al., 2015; Kim et al., 2016) support the likely generalizability of the present findings. ST131-*H3*0 was encountered across specimen types, indicating that it is a generalist, not just a UTI or sepsis-causing pathogen.

Within ST131, the observed temporal shifts in the relative abundance of the fluoroquinolone-resistant fraction (decreasing trend) and fluoroquinolone-susceptible fraction (increasing trend) and of each fraction’s three main pulsotypes are consistent with prior reports of ongoing clonal emergence and subsidence within the *E. coli* population (O'Neill et al., 1990; Manges et al., 2006; Smith et al., 2008; Johnson et al., 2019). The significant clinical and economic implications of such clonal shifts indicate a need for both ongoing monitoring and further study of its basis. For example, the observed decline in the fluoroquinolone-resistant fraction within ST131 may represent a salutary effect of decreasing fluoroquinolone prescribing in response to more stringent labeling and warnings. Notably, this shift within ST131 contrasts with an opposing trend observed elsewhere (Wang et al., 2021), evidence of the complexity and potentially locale-specific nature of resistance ecology.

The study’s second main finding was that ST1193, despite a modest overall prevalence (3%; less than multiple other STs), accounted for 11% of fluoroquinolone-resistant isolates, second only to *H*30. ST1193 has attracted attention over the past decade as an emerging disseminated multidrug-resistant pathogen, primarily of humans, but with spillover to domestic animals (Tchesnokova et al., 2018; Johnson et al., 2019; Kidsley et al., 2020; Pitout et al., 2022; Wyrsch et al., 2022). As with *H*30, both stepwise and mosaic evolution may have contributed to its epidemiologic success (Johnson et al., 2018). Understanding how these genetic changes contribute to the emergence of pathogens is critical for public health efforts.

The study’s third main finding from the fecal surveillance component was the 27% overall likelihood of detecting a subject’s clinical *E. coli* strain, including ST131-*H*30R and ST1193, within the individual’s fecal *E. coli* population at a time other than the infection episode. This supports the gut microbiota as an enduring reservoir for pathogenic *E. coli* strains (Schlaes et al., 1986; Maslow et al., 2004; Lautenbach et al., 2006; Johnson et al., 2008, 2016; Ulleryd et al., 2015; Overdevest et al., 2016). Because the likelihood of such detection varied significantly with the intensity and timing of fecal sampling, our 27% value doubtless is an underestimate. The intriguing observation that both of the ST1193-colonized subjects had matching ST1193 clinical isolates, while multiple *H*30-colonized subjects lacked a matching *H*30 clinical isolate, could indicate that ST1193 is more likely than *H*30 to cause infection when present in the gut, but a larger sample size would be needed to support this hypothesis.

The study’s fourth main finding was that repeat episodes were quite common, occurring in 31% of subjects and accounting for 41% of episodes, with the true values doubtless being higher, given the time-limited nature of our surveillance. Most repeat episodes (68%) involved the subject’s index strain. This is consistent with either relapse from a persisting endogenous focus, e.g., intra-epithelial bacterial colonies (Mulvey et al., 2000), or reintroduction of the strain from a persisting external reservoir, whether in/on the host, e.g., the gut or vaginal microbiota, or in the host’s environment, e.g., a colonized household member (Foxman et al., 2002; Czaja et al., 2009; Ulleryd et al., 2015; Johnson et al., 2016). Same-strain recurrences involved diverse specimen types and anatomical sites, pointing away from a unifying site-specific persistence mechanism for their occurrence, such as intra-urothelial IBCs.

The likelihood of recurrence and, specifically, same-strain recurrence diminished quite slowly over time: recurrences were documented up to 2,655d (7.3 years) after an initial episode, at which time 27% of recurrences still involved the index strain. This extends the known time for same-strain recurrence and provides a quantitative framework for assessing this phenomenon. It also supports the recommendation to include only one isolate per patient per year in cumulative susceptibility statistics, as a safeguard against clonal repeats (Cllinical Laboratory Standards Institute, 2022). Additionally, for patients with recurrent *E. coli* infections, it supports the potential utility of strain-specific secondary prevention measures, such as gut depopulation of the recurring strain, for example by using bacteriophage and/or probiotic *E. coli* (Galtier et al., 2016; Porter et al., 2022).

The fifth main finding was the association of specific bacterial characteristics—an ST131 (especially *H*30) or ST1193 genetic background, and fluoroquinolone resistance—with same-strain recurrences. Although this might indicate that such strains have an intrinsically greater propensity to cause recurrence, the largely neutral association of these characteristics with recurrence interval suggests otherwise. Possible alternate explanations include that such strains (i) preferentially colonize hosts who are predisposed to recurrences, or (ii) are able to persist in, on, or around the host longer than other strains, giving them more opportunity to cause infection. The former hypothesis is consistent with the known association of *H*30 with compromised hosts (Johnson et al., 2016), the latter with *H*30’s documented ability to out-persist other lineages as a gut colonizer in diverse host populations (Overdevest et al., 2016; Tchesnokova et al., 2020; Johnson et al., 2022). Discovery of the basis for this prolonged colonization phenotype conceivably could inform the development of mechanism-specific anti-colonization preventive measures.

Intriguingly, in multivariable analysis, fluoroquinolone resistance was the strongest independent predictor of the number of episodes, whereas *H*30 status (which tracks closely with fluoroquinolone resistance) reversed polarity to become a negative predictor, and ST1193 status lost statistical significance. These shifting associations likely reflect both the collinearity of these variables and the importance of resistance, genomic background, and their interaction in determining fitness. They also suggest that *H*30’s greater associated number of episodes may result not from *H*30 status *per se* but from the combination of an ST131 backbone and *H*30’s signature fluoroquinolone resistance.

The study has limitations, including the opportunistic (clinically driven) sampling; absence of epidemiologic and clinical data; reliance on PCR and PFGE-based strain typing rather than whole genome sequence analysis; single-site setting; and distinctive host population. It also has important strengths, including the extended (8-year) longitudinal surveillance, large study population (> 7000 clinical isolates), parallel attention to fecal colonization, and analysis of the temporal distribution of isolates in individual subjects, especially vis-a-vis bacterial characteristics and specimen type.

In summary, the recently emerged ST131-*H*30R lineage was the most abundant lineage overall among 7076 consecutive clinical *E. coli* isolates from veterans at the MVAHCS (2012–2019), followed—among fluoroquinolone-resistant isolates—by the emerging fluoroquinolone-resistant lineage ST1193. Both lineages had a prominent fecal reservoir that likely led to infections. Recurrent infections were common, often multiple, and usually same-strain (even years later), and involved diverse anatomical sites. ST131 (especially *H*30), ST1193, and fluoroquinolone-resistant strains generally were associated with multiple recurrences, but not with shorter recurrence intervals, consistent with host factors or prolonged persistence rather than greater aggressiveness explaining their clinical prominence.

## Data availability statement

The raw data supporting the conclusions of this article will be made available by the authors, without undue reservation.

## Ethics statement

The studies involving humans were approved by Minneapolis VA Health Care System Institutional Review Board. The studies were conducted in accordance with the local legislation and institutional requirements. The fecal sample donor participants provided their written informed consent to participate in this study.

## Author contributions

CC: Data curation, Formal analysis, Investigation, Methodology, Validation, Visualization, Writing – original draft, Writing – review & editing. PT: Data curation, Formal analysis, Methodology, Software, Validation, Visualization, Writing – review & editing. JJ: Conceptualization, Formal analysis, Funding acquisition, Investigation, Project administration, Resources, Supervision, Validation, Visualization, Writing – original draft, Writing – review & editing.
